# Supplementary material for: NMR spectroscopy reveals acetylsalicylic acid metabolites in the human urine for drug compliance monitoring
Source: PLoS One. 2021 Mar 8;16(3):e0247102. doi: 10.1371/journal.pone.0247102 (PMC7939264; doi:10.1371/journal.pone.0247102)
Supplement: Attachment — Submitted filename: PONE-D-20-12512_reviewer-3.pdf [file pone.0247102.s001.pdf]

PLOS ONE

Detection of acetylsalicylic acid metabolites in human urine for monitoring adherence to aspirin: the potential of NMR and SERS spectroscopy

--Manuscript Draft--

**Manuscript Number:**	PONE-D-20-12512
**Article Type:**	Research Article
**Full Title:**	Detection of acetylsalicylic acid metabolites in human urine for monitoring adherence to aspirin: the potential of NMR and SERS spectroscopy
**Short Title:**	Detection of acetylsalicylic acid metabolites in human urine: the potential of NMR and SERS spectroscopy
**Corresponding Author:**	Vladimir V Rafalskiy, PhD. MD<br>Immanuel Kant Baltic Federal University Institute of Medicine<br>Kaliningrad, Kaliningrad region RUSSIAN FEDERATION
**Keywords:**	acetisalicylic acid, metabolites, antiplatelet therapy, NMR spectroscopy, infrared spectroscopy, Raman spectroscopy
**Abstract:**	Background Cardiovascular disease (CVD) is the leading cause of morbidity and mortality worldwide. Long-term use of antiplatelet drugs is a well-studied therapy for the prevention of cardiovascular death. one of the important problems is compliance with lifelong administration of antiplatelet drugs, in particular acetylsalicylic acid (ASA). The aim of the study was to apply nuclear magnetic resonance and vibrational spectroscopy methods for the detection of patient compliance for long-term treatment of ASA. Methods and Findings Urine samples of volunteers were collected before and after taking 100, 300 and 3000 mg ASA for 24 h after dosing. Urine samples were examined by 1H NMR on a VARIAN 400 spectrometer in a magnetic field of 9 Tesla using a method of water signal suppression. Two-dimensional COSY experiment were performed with using of the following pulse sequence: (900) - t1 – (900) - t1. The 1H NMR profile of the spectrum of urine collected after 4 hours after taking 100 mg ASA shown significant changes -additional intense signals were observed in the 6.8-6.9 ppm and 7.7 ppm regions. New signals appear in the region of 6.8 ppm -7.1 ppm, which can be attributed to the main ASA metabolites – salicylic (SA) and salicyluric acids (SU). Thus, it was shown that the 1H NMR method is sensitive to the detection of low concentrations of ASA metabolites in the urine (at a dosage of 100 mg). Since the NMR method is not specific for certain substances, urine samples require preparation before carrying out of the experiments, adjusting the pH, and adding a deuterated solvent or standard for quantitative measurements and stabilization of experimental conditions. The prospects of Surface-Enhanced Raman Spectroscopy (SERS) realized on silver island films were shown for the detection of salicylic acid (SA) in patients' urine in case of taking more than 300 mg doze of acetylsalicylic acid. Characteristic markers have been revealed confirming the presence of SA metabolites both after taking 300 mg and 3000 mg of ASA. Skeletal molecular vibrations (C-C) at a frequency of 766 cm-1, C=O vibrations at 1637 cm-1 and characteristic maximums of C-O-H at 1152 cm-1 were detected. Certain perspectives for ASA overdose detection with IR-Fourier spectroscopy have been shown. The methodology for clear urine spectra detection has been performed. Conclusions The methods of detection of the main metabolites of ASA in urine have different efficiency. Detection of the main metabolites of ASA in the urine of the studied methods have different efficiencies. In particular, a method based on IR spectroscopy does not have sufficient sensitivity to detect the main metabolites of ASA. At the same time, methods based on 1H NMR and SERS can detect ASA metabolites (SA, SU) even after taking ASA at low doses. It is advisable to continue the study on based on NMR and SERS and to verify them with a wider range of doses of ASA and at different time intervals after administration of the drug.
**Order of Authors:**	Vladimir V Rafalskiy, PhD. MD
**Additional Information:**	
**Question**	**Response**

Powered by Editorial Manager® and ProduXion Manager® from Aries Systems Corporation

**Financial Disclosure**<br><br>Enter a financial disclosure statement that describes the sources of funding for the work included in this submission. Review the submission guidelines for detailed requirements. View published research articles from *PLOS ONE* for specific examples.<br><br>This statement is required for submission and **will appear in the published article** if the submission is accepted. Please make sure it is accurate.<br><br>**Unfunded studies**<br>Enter: *The author(s) received no specific funding for this work.*<br><br>**Funded studies**<br>Enter a statement with the following details:<br>• Initials of the authors who received each award<br>• Grant numbers awarded to each author<br>• The full name of each funder<br>• URL of each funder website<br>• Did the sponsors or funders play any role in the study design, data collection and analysis, decision to publish, or preparation of the manuscript?<br>• **NO** - Include this sentence at the end of your statement: *The funders had no role in study design, data collection and analysis, decision to publish, or preparation of the manuscript.*<br>• **YES** - Specify the role(s) played.<br><br>* typeset	The study was carried out with the financial support of the Russian Federal Property Fund and the Kaliningrad Region in the framework of the scientific project No. 19-415-390001
**Competing Interests**<br><br>Use the instructions below to enter a competing interest statement for this submission. On behalf of all authors, disclose any competing interests that could be perceived to bias this work—acknowledging all financial support and any other relevant financial or non-financial competing interests.<br><br>This statement **will appear in the published article** if the submission is accepted. Please make sure it is	NO authors have competing interests

accurate. View published research articles from *PLOS ONE* for specific examples.<br><br>**NO authors have competing interests**<br><br>Enter: *The authors have declared that no competing interests exist.*<br><br>**Authors with competing interests**<br><br>Enter competing interest details beginning with this statement:<br><br>*I have read the journal's policy and the authors of this manuscript have the following competing interests: [insert competing interests here]*<br><br>* typeset	
**Ethics Statement**<br><br>Enter an ethics statement for this submission. This statement is required if the study involved:<br><br>• Human participants<br>• Human specimens or tissue<br>• Vertebrate animals or cephalopods<br>• Vertebrate embryos or tissues<br>• Field research<br><br>Write "N/A" if the submission does not require an ethics statement.<br><br>General guidance is provided below. Consult the submission guidelines for detailed instructions. **Make sure that all information entered here is included in the Methods section of the manuscript.**	Urine samples were collected from healthy volunteers after signing informed consent. Informed consent, study protocol was approved by an independent ethics committee - Local ethic committee of Clinical Trials Center of Immanuel Kant Baltic Federal University, A.Nevskogo St. 14, Kaliningrad, Russia.

Powered by Editorial Manager® and ProduXion Manager® from Aries Systems Corporation

**Format for specific study types**<br><br>**Human Subject Research (involving human participants and/or tissue)**<br>• Give the name of the institutional review board or ethics committee that approved the study<br>• Include the approval number and/or a statement indicating approval of this research<br>• Indicate the form of consent obtained (written/oral) or the reason that consent was not obtained (e.g. the data were analyzed anonymously)<br><br>**Animal Research (involving vertebrate animals, embryos or tissues)**<br>• Provide the name of the Institutional Animal Care and Use Committee (IACUC) or other relevant ethics board that reviewed the study protocol, and indicate whether they approved this research or granted a formal waiver of ethical approval<br>• Include an approval number if one was obtained<br>• If the study involved *non-human primates*, add *additional details* about animal welfare and steps taken to ameliorate suffering<br>• If anesthesia, euthanasia, or any kind of animal sacrifice is part of the study, include briefly which substances and/or methods were applied<br><br>**Field Research**<br><br>Include the following details if this study involves the collection of plant, animal, or other materials from a natural setting:<br>• Field permit number<br>• Name of the institution or relevant body that granted permission	
**Data Availability**<br><br>Authors are required to make all data underlying the findings described fully available, without restriction, and from the time of publication. PLOS allows rare exceptions to address legal and ethical concerns. See the PLOS Data Policy and FAQ for detailed information.	Yes - all data are fully available without restriction

A Data Availability Statement describing where the data can be found is required at submission. Your answers to this question constitute the Data Availability Statement and **will be published in the article**, if accepted.<br><br>**Important:** Stating 'data available on request from the author' is not sufficient. If your data are only available upon request, select 'No' for the first question and explain your exceptional situation in the text box.<br><br>Do the authors confirm that all data underlying the findings described in their manuscript are fully available without restriction?	
**Describe where the data may be found in full sentences. If you are copying our sample text, replace any instances of XXX with the appropriate details.**<br><br>• If the data are **held or will be held in a public repository**, include URLs, accession numbers or DOIs. If this information will only be available after acceptance, indicate this by ticking the box below. For example: *All XXX files are available from the XXX database (accession number(s) XXX, XXX.).*<br>• If the data are all contained **within the manuscript and/or Supporting Information files**, enter the following: *All relevant data are within the manuscript and its Supporting Information files.*<br>• If neither of these applies but you are able to provide **details of access elsewhere**, with or without limitations, please do so. For example:<br><br>*Data cannot be shared publicly because of [XXX]. Data are available from the XXX Institutional Data Access / Ethics Committee (contact via XXX) for researchers who meet the criteria for access to confidential data.*<br><br>*The data underlying the results presented in the study are available from (include the name of the third party*	All relevant data are within the manuscript and its Supporting Information files

and contact information or URL).<br>• This text is appropriate if the data are owned by a third party and authors do not have permission to share the data.<br><br>* typeset	
Additional data availability information:	

Manuscript Click here to access/download;Manuscript;renamed_d36e7.pdf

Detection of acetylsalicylic acid metabolites in human urine for monitoring adherence to aspirin: the potential of NMR and SERS spectroscopy

Vladimir Rafalskiy[1], Galina Kupriyanova[2], Andrey Zyubin[3], Ivan Mershiev[2], Karina Matveeva[3], Ekaterina Moiseeva[1], Elizaveta Demishkevich[3], Ilia Samusev[3]

1 - Medical Institute, Center for Clinical Trials; 2 - Laboratory of magnetic resonance methods in substance research; 3 - REC «Fundamental and Applied Photonics. Nanophotonics»
Immanuel Kant Baltic Federal University, A.Nevskogo St. 14, Kaliningrad, Russia

Corresponding author information: Vladimir Rafalskiy, v.rafalskiy@mail.ru
Md, PhD, professor of medical institute Immanuel Kant Baltic Federal University

Abstract

Background

Cardiovascular disease (CVD) is the leading cause of morbidity and mortality worldwide. Long-term use of antiplatelet drugs is a well-studied therapy for the prevention of cardiovascular death. one of the important problems is compliance with lifelong administration of antiplatelet drugs, in particular acetylsalicylic acid (ASA). The aim of the study was to apply nuclear magnetic resonance and vibrational spectroscopy methods for the detection of patient compliance for long-term treatment of ASA.

Methods and Findings

Urine samples of volunteers were collected before and after taking 100, 300 and 3000 mg ASA for 24 h after dosing. Urine samples were examined by 1H NMR on a VARIAN 400 spectrometer in a magnetic field of 9 Tesla using a method of water signal suppression. Two-dimensional COSY experiment were performed with using of the following pulse sequence: (900) - t1 – (900) - t1. The 1H NMR profile of the spectrum of urine collected after 4 hours after taking 100 mg ASA shown significant changes -additional intense signals were observed in the 6.8-6.9 ppm and 7.7 ppm regions. New signals appear in the region of 6.8 ppm -7.1 ppm, which can be attributed to the main ASA metabolites – salicylic (SA) and salicyluric acids (SU). Thus, it was shown that the 1H NMR method is sensitive to the detection of low concentrations of ASA metabolites in the urine (at a dosage of 100 mg). Since the NMR method is not specific for certain substances, urine samples require preparation before carrying out of the experiments, adjusting the pH, and adding a deuterated solvent or standard for quantitative measurements and stabilization of experimental conditions. The prospects of Surface-Enhanced Raman Spectroscopy (SERS) realized on silver island films were shown for the detection of salicylic acid (SA) in patients' urine in case of taking more than 300 mg doze of acetylsalicylic acid. Characteristic markers have been revealed confirming the presence of SA metabolites both after taking 300 mg and 3000 mg of ASA. Skeletal molecular vibrations (C-C) at a frequency of 766 cm-1, C=O vibrations at 1637 cm-1 and characteristic maximums of C-O-H at 1152 cm-1 were detected. Certain perspectives for ASA overdose detection with IR-Fourier spectroscopy have been shown. The methodology for clear urine spectra detection has been performed.

Conclusions

The methods of detection of the main metabolites of ASA in urine have different efficiency. Detection of the main metabolites of ASA in the urine of the studied methods have different efficiencies. In particular, a method based on IR spectroscopy does not have sufficient sensitivity to detect the main metabolites of ASA. At the same time, methods based on 1H NMR and SERS can detect ASA metabolites (SA, SU) even after taking ASA at low doses. It is advisable to continue the study on based on NMR and SERS and to verify them with a wider range of doses of ASA and at different time intervals after administration of the drug.

1. INTRODUCTION

Cardiovascular disease (CVD) is the leading cause of morbidity and mortality among individuals worldwide. In 2017, CVD caused an estimated 17.8 million deaths worldwide, corresponding to 330 million years of life lost and another 35.6 million years lived with disability (1, 2). For high-risk patients it is important that treatment is continuous and that good compliance is maintained over the long term (3).

Currently, several approaches have been developed and applied in assessing the adherence to aspirin: methods for counting the drug, interviewing patients, evaluating platelet function, determining aspirin metabolites in the blood and urine. Only a few studies measured levels of acetylsalicylic acid (ASA) metabolites in patients with CVD taking ASA for CVD prevention (4, 5).

The biological half-life of ASA is about only 20 min (6) and ASA undergoes rapid hydrolysis by esterases in the gastric mucosa, liver, plasma and erythrocytes, producing major ASA metabolites – salicylic acid (SA) and salicyluric acid (SU) as far as minor metabolites – gentisic acid, salicyl-acyl glucuronide, salicylphenolic glucuronide, salicyluric acid phenolic glucuronide and gentisuric acid (7-9). The urinary excretion ratio of the three main metabolites of ASA in human urine – SA, SU and gentisic acid is 8.3: 45.0: 1.0 (9). Therefore, the most rational approach for monitoring ASA ingestion is to identify two main ASA metabolites (SA and SU) in the blood or in the urine.

Various methods, such as HPLC and LC–MS/MS, have been developed to determine ASA and ASA metabolites in human blood and urine (9, 10). Therefore, there are no researches focused on ASA analysis of urine metabolites related to patient's compliance monitoring process. The aim of this study was to study the possibilities of using vibrational spectroscopy methods (SERS and IR spectroscopy) and nuclear magnetic resonance (NMR) spectroscopy to identify ASA metabolites for monitoring patients for compliance with ASA.

2. METHODS

2.1 Human investigation

The investigation used a panel of healthy volunteers, drawn from the students and staff of Immanuel Kant Baltic Federal University, ages 19-50 years, body wait 55-85 kg. The subjects had taken no drugs for at least seven days before their participation in the study; no other exclusion criteria were applied. Urine samples were collected from healthy volunteers after signing informed consent. Informed consent, study protocol was approved by an independent ethics committee.

Urine samples of volunteers were collected before and after taking 100, 300 and 3000 mg ASA for 24 h after dosing. Freshly collected urine was centrifuged at 2000 g, after which the supernatant was collected and passed through a filter nozzle (average pore diameter is 0.2 μm, membrane diameter is 28 mm, cellulose acetate, fiberglass (Merck, Millipore)). Then, using a buffer solution, the urine pH was adjusted to 7.2-7.4. The volume and pH of the sample was recorded at the time of collection, and samples stored at -20°C until analyzed.

2.2. NMR spectroscopy study of the urine samples

540 μl of prepared and pH-corrected urine sample was placed in standard 5 mm sample tube, then 60 μl of D_2O were added to the sample as a reference frequency standard. Urine samples were examined by ^{1}H NMR on a VARIAN 400 spectrometer in a magnetic field of 9 Tesla. The main parameters of the one-dimensional experiments were as follows: the duration of 90^0 pulses was 2.5 μs, the number of scans was varied from 128 to 8192. All spectra were acquired at 25° C temperature. All chemical shifts were determined by the signal of the residual protons of deuterated water. The spectral assignment of signals of endogenous compounds in urine samples was performed by taking into account the literature data of chemical shifts in biological fluids, spin-spin interaction constants. ^{1}H NMR spectra of three standard acids: salicylic, salicyluric and gentisic acids, corresponding to the major metabolites of acetylsalicylic acid, obtained from Sigma-Aldrich, were acquired under the same experimental conditions as urine samples. Two-dimensional COSY experiment were performed with using of the following pulse sequence: (90^0) - t_1 – (90^0) - t_1.

To suppress water signal, various pulse sequences have been tested (WATER suppression by GrAdient Tailored Excitation scheme - WATERGATE). The WATERGATE technique turned out to be the most effective for recording one-dimensional spectra of the studied samples. It allows us to obtain spectra with minimal artifacts in the region of the suppressed water signal. WATERGATE makes it possible to observe signals from protons whose frequencies are close to or overlap with the water resonance (H[illegible]HO). The WATERGATE is a spin echo sequence using frequency-selective excitation pulses with a minimum excitation power at the frequency of the water signal. Fine tuning of the sequence parameters was carried out based on the effectiveness of the water signal suppression. To identify the metabolites, a two-dimensional COSY pulse sequence was used to reveal the proton-proton interactions of the overlapping signals in the area of interest. In two-dimensional spectra, the water signal was suppressed by the WET method.

Preliminarily, in order to distinguish the signals of ASA metabolites against the background of healthy human metabolites, such as hippuric acid, we recorded spectra of SA, SU and gentistic acids with the addition of deuterated water, and determined chemical shifts relative

to TMS. Urine samples from healthy volunteers before and after taking 100 mg, 300 mg and 3000 mg ASA, prepared as described above, were studied by ^{1}H NMR using a method of water signal suppression.

2.3. Surface-enhanced Raman spectroscopy study of the urine samples

2.3.1. Silver nanoparticles synthesis

A wet chemistry chemical method based on the reduction of silver ions with sodium tetrahydride borate (LTD «Lenreactiv», Russia; Purity > 98%) was used for silver nanoparticles (NPs) synthesis. To perform the synthesis, 100 ml of Milli-Q water (Ω = 18,2) and 7.5 mg of $NaBH_4$ was added in chemically purified flat-bottomed flask. Then the flask was placed on magnetic stirrer, where the resulting solution was subjected to cooling in ice to a temperature t = 0° C and mixing with a ferrite anchor (~1100 rpm). After that, 5 ml of $AgNO_3$ with a concentration of 10^{-3} M was quickly added dropwise into the solution. The reduction reaction of silver ions led to the formation of nanoparticles and a change in the color of the solution from colorless to yellow-brown. The suspension was kept at zero temperature for another 20 minutes to perform the full silver reduction. After that, a freshly prepared hydrosol of nanoparticles was used in 2 hours for SERS experiments.

2.3.2. Optical properties of silver nanoparticles AgNPs

To study the optical properties and sizes of silver nanoparticles, spectrophotometry and photon-correlation spectroscopy were used. The absorption spectrum (Fig. 1b) was obtained using a UV-2600 double-beam spectrophotometer (Shimadzu, Japan). To determine the size of the nanoparticles (Fig. 1a), the Photocor Complex analyzer (Photocorr, Russia) was used. The calculation of the concentration of nanoparticles in the obtained hydrosols was carried out according to the formula:

$$C = \frac{3m}{4\pi r^3 \rho N_a V} \quad , \quad (1)$$

where m is mass of silver in the solution ($m = 3.397$ mg); r is NPs radius ($r \approx 12$ nm); ρ is silver density ($\rho = 10.49\ \mathrm{g/cm^3}$), V is solution volume (V=0.12 l). The resulting concentration of silver spherical nanoparticles was $C=6{,}19\cdot 10^{-10}$ M.

2.3.3. SERS substrates preparation

Since Raman spectroscopy experiments demonstrated small cross-sections of scattering, the SERS active surfaces were prepared in order to amplify the signal of Raman scattering. High quality quartz glasses were used and silver nanoparticles were thermally deposited on it. Quartz glasses were chemically cleaned with isopropyl alcohol, after which they were placed on a pre-heated tile. The glass heating temperature was 40° C. Temperature control was carried out using an external thermal controller. A created hydrosol of silver nanoparticles was applied dropwise (5 μl) to the prepared quartz glass. The next 5 μl drop was added on the glass after the first droplet

dried. The third 5 μl drop was added on the layer after the second drop was dried. As a result the silver clusters were formed on the substrate surface. Modified substrates with silver nanoparticles were ready for used for SERS experiment in 10 min after drying of the hydrosol third layer. The effective amplification of the Raman signal reached orders of magnitude 10^2 – 10^3 раз for urine samples.

2.3.4. Registration of Raman spectra of the urine samples

Raman spectra of the urine samples were obtained using Raman Centaur U (LTD «NanoScanTechnology», Russia) spectrometer with spectral resolution 2,5 cm^{-1}. The spectrometer was equipped with 532 nm Cobolt Samba DPSS laser (50mW) which was applied to register the SERS spectra. The optical scheme of the spectrometer included Olympus BX41 microscope (Olympus, Japan) with 100X (NA 0.9) objective to perform beam positioning and scattered photons collection. Spectrometer M266 (SolarLS, Belarus) monochromator had a focal length of 284 mm, 1200 g/mm holographic diffraction grating and was equipped with a 1024 × 256 pixels CCD thermoelectric cooled detector (Andor Tech., UK). Urine samples were put on the chemically purified quartz glass and placed in the microscope holder. The laser beam was positioned on the sample located on silver cluster USB Video Camera (Olympus, Japan) and the laser spot from the Raman spectrometer was focused on the sample's surface. The size of the laser spot was varied from 1 × 25 μm to 1 × 30 μm depending on laser power used. To prevent sample destruction and achieve the best signal-to-noise ratio, laser power embedded to the sample was manually adjusted from 5 to 50 mW for DPPS lasers. The notch filters were applied to eliminate Rayleigh scattering. Three times averaged spectra from one place of the droplet have been collected for each sample. Signal acquisition time was 50s. Acquisition time and number of averages were selected manually to get a best signal-to-noise ratio. Raman spectra were recorded in a wavenumber «fingerprint» range for organic samples between 600 cm^{-1} and 1800 cm^{-1} with spectral resolution 2.5 cm^{-1}. The instrument was calibrated before assessment of each series of specimens with silicon standard (Horiba, Japan) at a static spectrum centered at 520.1 cm^{-1} for 1 s. All spectral data was saved after the registration as .txt files for further assessment.

BioRad-KnowItAll Informatics System (Thermo Fisher Scientific Inc., USA) was used for manual linear baseline correction and Savitzky–Golay filtering. All spectra were baseline corrected and normalized to the maximum signal intensity.

2.4. Infrared (IR)-Fourier spectroscopy of urine samples in KBr pellets

Urine samples were subjected to a single freeze after collection. Before the experiment, the clinical samples were uniformly thawed in a water bath at room temperature (T = 21 °C). After complete thawing, the samples in the tubes were inverted 4 times by 180° for 3 seconds. 20 μl of urine was taken with an Eppendorf Research+ microliter mechanical pipette and deposited on the

on the walls of an agate mortar to form a thin layer. Mortar was purified with isopropyl alcohol before the experiment. After it, the mortar was placed into desiccator and heated to 60° C. Evaporation of urine occurred within 20 minutes. After evaporation, traces of remained urine formed of a semi-matte white coating on the mortar walls. The coating was thoroughly cleaned from the walls of the mortar and then 0.25 grams of an optically transparent and chemically pure KBr powder (Pike Tech., UK; purity > 99%) was added to it. The resulting mass was carefully mixed with a pestle in order to achieve a finely divided state of the mixture. Evenly distributing the mixture on the inside of the mortar, the sample was again drying at 60° C in a desiccator for 20 minutes. After that, the grinding and drying procedure was repeated under the same conditions with the initial amount of the sample. The next step was the pressing of a KBr pellet from the resulting mixture with a hydraulic press at a pressure of 7-7.5 tons on sample within three minutes. After of the procedures, the fully transparent tablet was carefully removed from the press holder and placed at the spectrometer holder. The spectrum was recorded on a Prestige-21 IR-Fourier spectrometer (Shimadzu, Japan). Spectra were recorded in the characteristic region of 400-3100 cm^{-1}.

3. RESULTS

3.1. Detection of ASA metabolites by NMR methods

^{1}H NMR fragments of the spectra of a volunteer urine samples collected after taking 100 mg and 300 mg of the drug are shown in Fig. 1,2. In order to stabilize the resonance conditions, 3-(trimethylsilyl) -2,2,3,3-tetradeuteropropionic acid (TSP-d4) (Euriso-top) was used as the internal standard of the internal chemical shift reference. Samples were adjusted to pH = 7.2. In this paper, the assignment of signals is given relative to TMS.

Analysis of the 1H NMR spectra of urine samples of healthy volunteers before and after taking aspirin have showed different dynamics of metabolism depending on the time of collection of urine. There were no significant changes in the spectra of urine samples collected after 1.5 hours of taking medication (Fig. 1(a)). A slight line shift can be caused by a change in the pH of the sample. However, the ^{1}H NMR profile of the spectrum of urine collected from volunteers after 4 hours from taking 100 mg of the drug undergoes significant changes. In the spectrum of the volunteer urine sample, collected after 4 hours from taking 100 mg of the drug, additional intense signals were observed in the 6.8-6.9ppm and 7.7 ppm regions, overlapping with hippuric acid signals. In order to identify the signals, spectra of urine samples were compared with the spectra of hippuric and salicylic acids. In fig. 2 (a, b) it can be seen that the line intensity in the region of 7.7 ppm - 7.3 ppm changes and new signals appear in the region of 6.8 ppm -7.1 ppm, which can be attributed to the main ACA metabolites - SA and SU.

More pronounced changes in the ^{1}H NMR spectra can be observed for urine samples, collected after 4 hours of patient taking 100 mg single-dose aspirin (Fig. 3). In the region of 6.7 ppm - 6.8 ppm one can observe intense overlapping signals of SA and SU acids, as well as a clearly distinguishable triplet and doublet of salicylic acid in the regions of 7.15–7.2 ppm and 7.60 –7.65 ppm.

In order to unambiguously assign signals and identify metabolites, correlation spectra of COSY were recorded. Comparison of two-dimensional spectra of urine before (red) and after (green) reception of 3g of ASA are shown Fig. 4. Cross peaks from the signals of SA and SU acids are observed in the regions of 7.6, 7.25, 6.75 ppm. Correlation peaks uniquely indicate the presence of coupled signals of the interacting protons of the benzene ring of salicylic acid. Thus, the study showed that the spectral region is 6.7 ppm - 6.8 ppm [illegible]n which there are no noticeable signals in the urine of a healthy person, can be used to establish diagnostic signs of taking medications. Changes in this region of the urine spectrum are present whether the volunteer takes 3000 mg, 300 mg or 100 mg of the drug.

3.2. SERS analysis of ASA metabolites

To analyze the data obtained by the results SERS experiments, the Raman spectra of the main metabolites of ASA were registered: SA (Sigma, purity > 98%), hippuric acid (Sigma, purity > 98%), gentisic acid (Sigma, purity > 98%). The positions of the main characteristic maxima for each metabolite were identified. To record reference spectra, 1 mg of pure substance in the crystalline phase was taken. Further, a spectral analysis was carried out to identify spectral differences in the case after taking 300 mg and 3000 mg of ASA and comparing it with the main metabolites of ASA.

According to the results of the SERS experiment of clinical samples, characteristic bands were revealed that confirm the presence of SA metabolites both after taking 300 mg of ASA and after taking 3000 mg of ASA. In the case of receiving 300 mg, characteristic vibrational bands of skeletal molecular vibrations (C-C) at a frequency of 766 cm^{-1}, C = O vibrations at a frequency of 1637 cm^{-1}, which are the part of SA molecules, were recorded. In the spectral frequency range of 1100 - 1200 cm^{-1}, characteristic maximums of C-O-H vibrations in SA were detected at a frequency of 1152 cm^{-1}. In the control sample without ASA, either the absence of such a vibrational band or the presence of characteristic non-intense shoulder in the spectrum at a frequency of 1152 cm^{-1} was observed (Fig. 4). In such a control sample, characteristic vibrations of NH_2 urea at a frequency of 1159 cm^{-1} were also recorded (11-15).

In the case of taking a subtoxic dose of 3000 mg of ASA, an increase in the intensity of the recorded vibrations of the C-O-H and C=O groups at frequencies of 1637 cm^{-1}, 1152 cm^{-1} was

registered compared with the case of taking 300 mg of ASA. On the other hand, the intensity of the vibrational band for a frequency of 766 cm^{-1} decreased, which may be correspond to repeatability problems of SERS signal (16). In this regard, the authors prioritize interpretation of the results of SERS for identified spectral shifts, while a change in intensity is a secondary sign. Table 1 shows the positions and intensities of the main characteristic maxima.

.

3.3 Determination of ASA metabolites by Fourier-transform infrared spectroscopy (FTIR)

To analyze the obtained data from the results of FTIR spectroscopy experiments, the obtained spectra of the samples after taking 300 mg and 3000 mg of ASA and control were correlated with the spectra of the studied metabolites. The experiments showed the lack of coincidence of the studied bands with the following metabolites: SA, SU, hippuric acid, gentisic acid. Fig. 8 shows the obtained spectra for the studied metabolites. Spectral differences in the case of taking a variable dose of ASA and in the case of control were not found, as well as spectral correlation with the characteristic maxima of metabolites.

During the experiment, 1 mg of SA standard was added to 20 µl of a urine sample without ASA. In this case, low-intensity peaks of SA metabolite in the sample were detected. Since the detection of metabolites was demonstrated by combining FTIR spectroscopy with other methods (17, 18) the authors believe that the concentration of metabolites even in case of receiving a subtoxic dose of 3000 mg is insufficient for detection of SA in urine, however, there are certain prospects for the use of FTIR spectroscopy for studies overdoses of ASA are available

4. DISCUSSION

Long-term therapy with low-dose (75-325 mg) ASA, is highly effective for the secondary prevention of cardiovascular events. Low-dose aspirin (LDA) also confers a 10% risk reduction for pre-eclampsia and a 20% risk reduction for fetal growth restriction in high-risk pregnant women, (5, 19-21). Poor compliance with low-dose ASA therapy ranged from approximately 10% to over 50%, and patient-initiated discontinuation of therapy occurred in up to 30% of patients (22). Nonadherence or discontinuation of aspirin imparted a 3-fold increased risk of overall thrombotic events and a 2-fold increase in the risk of coronary artery disease and coronary artery bypass grafting (23).

Non-invasiveness and the ability to collect a large amount of quantitative and qualitative information, in particular about the biochemical composition, conformations of molecules in biological fluids, the relative simplicity of sample preparation, and high analysis speed are potential advantages FT-IR and Raman spectroscopy (including SERS) methods for determining ASA metabolites.

The NMR method is becoming increasingly widespread as an experimental basis for studying the metabolic profile of humans and animals during their life activity under normal conditions and various pathologies, as well as under the influence of drugs (24), and is an indispensable method for identifying metabolites. The prospects of using ^{1}H NMR spectroscopy in studying the composition of urine in order to develop new laboratory methods for diagnosing diseases were shown in (25). It was noted (24) that ^{1}H NMR spectroscopy is an express method for the determination of various metabolites in biological fluids, such as urine and blood. In papers (25, 26), ^{1}H NMR was proposed as a convenient method for detecting salicylate poisoning by detecting the main metabolites of acetylsalicylic acid in saline samples: salicylic acid (SA), gentisic acid, and salicyluric acid (SU). In (25), the NMR frequencies and spin – spin coupling constants of SA and SU were determined by two-dimensional J-resolved spectroscopy.

In a recently published review, an attempt was made to systematize existing methods for the qualitative and quantitative study of adherence to aspirin. It turned out that the use of objective quantitative tests based on laboratory or instrumental data in research is extremely limited. In particular, it was shown that only 6% of researchers relied on an assessment of serum or urinary thromboxane. And methods based on measuring levels of aspirin metabolites are mentioned in only 2 of 87 studies (27). In our opinion, one of the explanations why the methods based on the detection of aspirin metabolites are not widespread is their complexity and high cost. First of all, this concerns various modifications of the HPLC. The physical methods investigated in this work for identifying aspirin metabolites are largely devoid of these drawbacks.

In our study volunteer urine samples were evaluated before and after receiving of 300 mg and 3000 mg ASA. There were revealed significant changes in the ^{1}H NMR spectra associated with the presence of additional ASA metabolite signals. The complexity of the unambiguous identification of emerging metabolites is due to the fact that the ^{1}H NMR spectrum of a healthy person's urine contains a number of metabolite signals whose chemical shifts are in the range from 6.9 ppm to 8 ppm (32, 33) related to the signals of aromatic protons of hippuric acid and phenols. A comparative analysis of the spectra of urine samples and the spectra of pure forms of hippuric, SA and SU acids dissolved in water made it possible to identify the presence of metabolites of SA and SU acids. Two-dimensional correlation (COSY) experiments confirmed the appearance of signals of SA and SU metabolites in urine samples of volunteers receiving aspirin by detecting additional cross peaks whose chemical shifts are in the range from 6.9 ppm to 8 ppm, which is consistent with the results of (32-34, 40). After receiving high dosed of aspirin (3000 mg), changes in the ^{1}H NMR spectrum of the urine were detected not only in regions characteristic of the identification of ASA metabolites, but also in the region of the spectrum characteristic of urea identification - 5.3-5.8 ppm. It was revealed that the peak intensity in the spectral region of 5.3–

5.8 ppm, characteristic of urea, two hours after administration of ASA at a dose of 3000 mg, was lower than before administration of ASA (Fig. 3 b).

This fact can be explained by the peculiarities of the pharmacological action of ASA. Nephrotoxicity of non-steroidal anti-inflammatory drugs (NSAIDs) as well as ASA has been widely described (28). ASA nonselective inhibit renal cyclooxygenases in the arachidonic acid pathway, this disrupts the production of prostaglandins which results in renal vasoconstriction (29, 30) and decrease urea glomerular filtration rate.

The NMR method was sensitive to the detection of low concentrations of ASA metabolites in the urine (at a dosage of 100 mg).

However, not all volunteers showed changes in the ^{1}H NMR spectra of urine samples after receiving small doses of the drug. It was found that the intensity of the signals of ASA metabolites in the samples depends on the time of urine collection, which passed after administration of ASA. The problem of the individual metabolic rate of ASA in volunteers is still waiting for its solution. Moreover, a comparison was made of the amount of salicylic acid (SU) and salicylic acid (SA) daily excreted in the urine by non-vegetarians and vegetarians who do not take salicylate drugs, and patients taking low doses of aspirin (75 or 150 mg aspirin / day) (31). The authors found that vegetarians daily released significantly more SU (4.98–26.60 μmol / 24 hours) than non-vegetarians (0.87–12.23 μmol / 24) hours), although the amounts were significantly lower than those that are secreted by patients taking aspirin. The amount of SA, relative to SU, excreted from the body in all four studied groups was relatively low, which probably reflects the number of metabolic pathways that SA can use. This fact can also be used to establish the identification of low-dose aspirin.

Thus, it was shown that the ^{1}H NMR method is sensitive to the detection of low concentrations of ASA metabolites in the urine (at a dosage of 100 mg). The probability of detecting ASA metabolites in the urine depends on the interval from taking the drug to collecting tests and, possibly, the individual metabolic rate of the patient. Since the NMR method is not specific for certain substances, urine samples require preparation before carrying out of experiments, adjusting the pH, and adding a deuterated solvent or standard for quantitative measurements and stabilization of experimental conditions. Nevertheless, the sensitivity of the method allows the detection of ASA metabolites in therapeutic doses used for the prevention of cardiovascular diseases.

Raman and FTIR spectroscopy are highly versatile techniques and have minimum sample preparation. Samples can be simply placed onto different surfaces (ZnSe crystals, quartz, SERS active surfaces e t.c.) for fast and label-free analysis. Combinations of complementary Raman and FTIR methods can avoid researchers to get away from FTIR sample absorption in water (using

Raman instead) and allowing usage of suitable FTIR methods as attenuated total reflectance (ATR), analysis in KBr pellets. The rapidity of spectral collection for both methods is an advantage and more than 800 spectra can be collected within 24 h using both methods.

Raman spectroscopy is widely used for diagnostic applications (32-34). For urea analysis, Raman spectroscopy is applied to detect and monitor nitrogen compounds in case of kidney deceases (35). Authors (36) perform glucose identification in diluted urine by the Raman spectroscopy method. Urine metabolites can be detected for physically active subjects (37). To amplify Raman scattering low cross-sections for urine metabolites detection SERS-based methods towards plasmonic nanoparticle colloids (38) and surfaces (16) are used for it. SERS can be effectively used for detection the presence of illegal substances in sportsmen's urine (39, 40). Paper (41) demonstrated prospects of SERS spectroscopy for multiplex detection of two families of urine metabolites correlated with miscarriage risks: 5β-pregnane-3α, 20α-diol-3α-glucuronide and tetrahydrocortisone. IR spectroscopy is also used for metabolites detection, but mostly in combination with other methods: HPLC spectroscopy (17) and liquid chromatography-mass spectrometry (LC-MS) (42). The detection of lapatinib metabolites in liver tissue can be performed by mass spectrometry imaging using infrared matrix-assisted laser desorption electrospray ionization (IR-MALDESI) (18). The metabolic fingerprinting using FTIR are undertaken using NIR measurement of lactate in human blood (43) and metabolites in faeces (44).

Thus, the comprehensive study has showed that the detection of the main metabolites of ASA in the urine of the studied methods have different efficiencies. In particular, a method based on IR spectroscopy does not have sufficient sensitivity to detect the main metabolites of ASA. At the same time, methods based on 1H NMR and RS / SERS can detect SA, SU acids even after taking ASA at low doses used to prevent cardiovascular disease. It is advisable to continue the study on based on NMR and RS / SERS and to verify them with a wider range of doses of ASA and at different time intervals after administration of the drug.

5. Acknowledgments

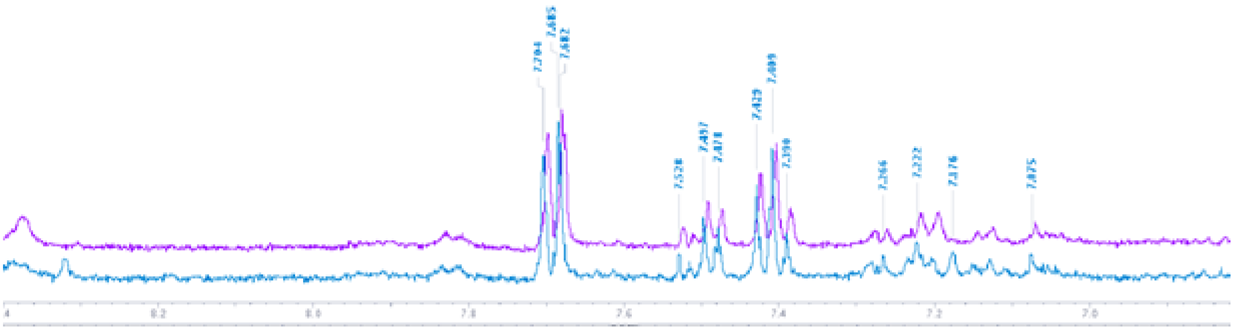

a)

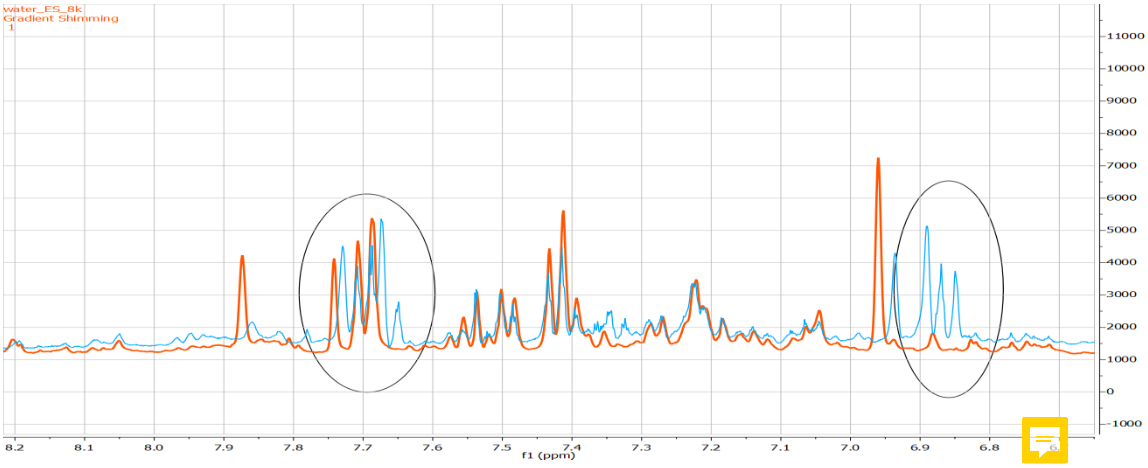

b)

Fig. 1. ^{1}H NMR of a urine sample from a healthy volunteer N3 (red), and ^{1}H NMR spectrum collected after 90 minutes (blue) (a) . ^{1}H NMR of a urine sample from a healthy volunteer M (orange), and ^{1}H NMR spectrum collected after 4 hours after taking aspirin (blue)

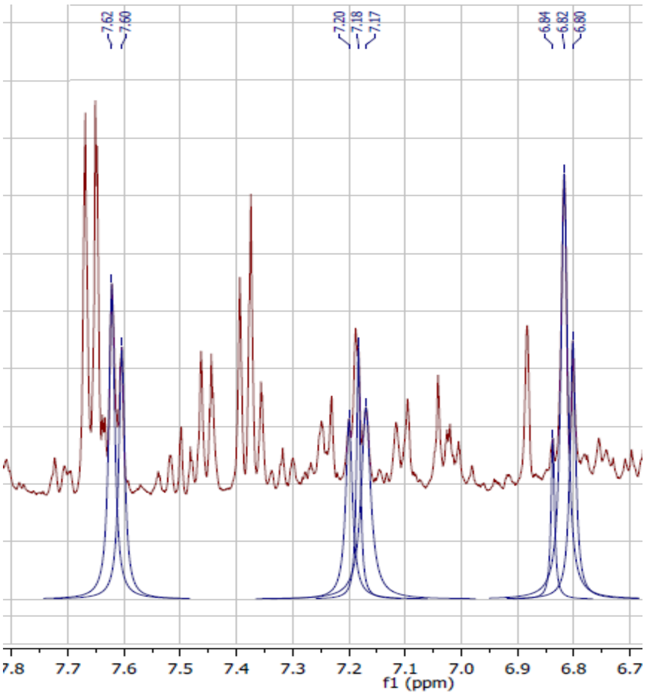

a)

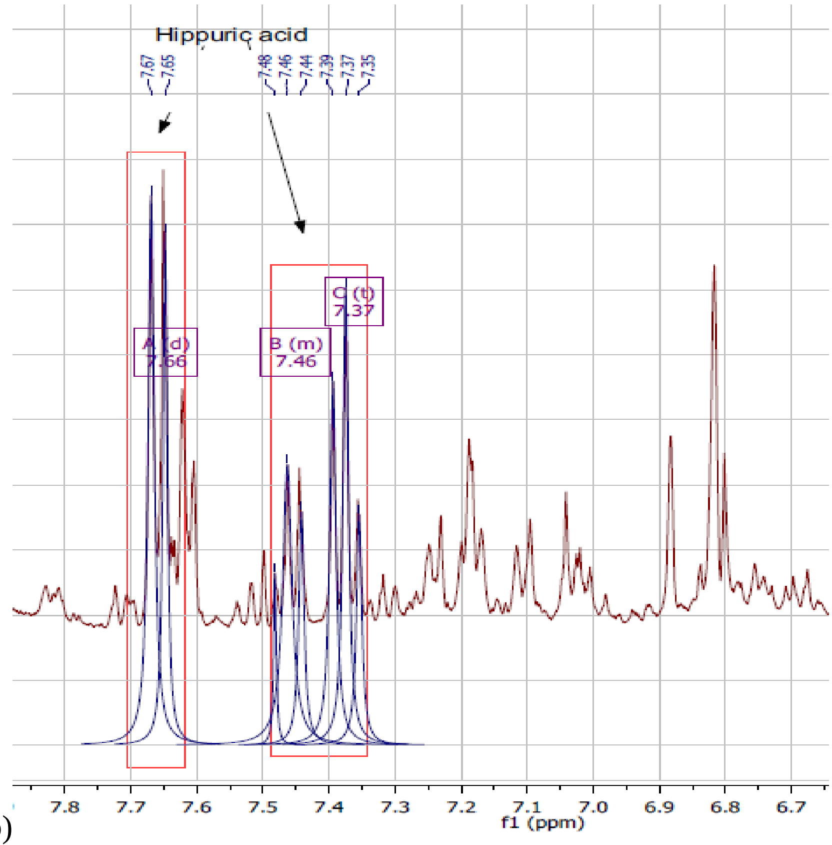

b)

Figure 2: ^{1}H NMR fragment of the spectrum of a urine sample collected after taking 100 mg of the drug. The spectrum is compared with the spectra of salicylic and salicylic acid (a) and with the spectrum of hippuric acid (b).

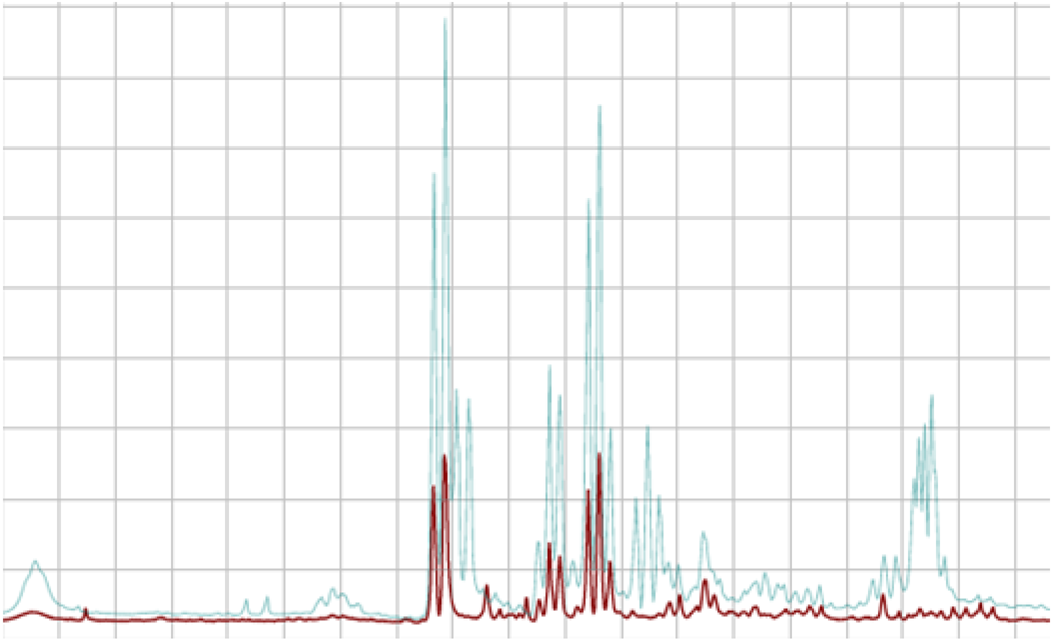

a)

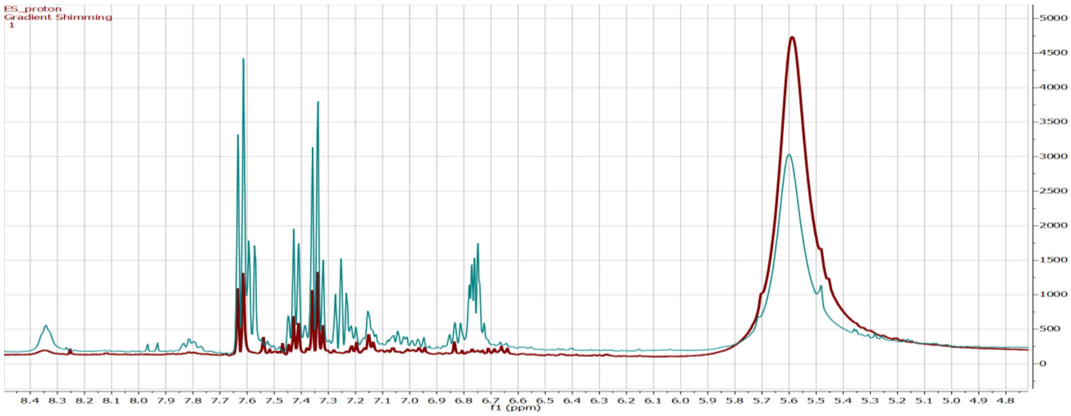

b)

Figure 3: ^{1}H NMR fragments of the spectrum (a,b) of a volunteer urine sample collected before (red) and after 2 hours after taking 3000 mg of the ACA (green).

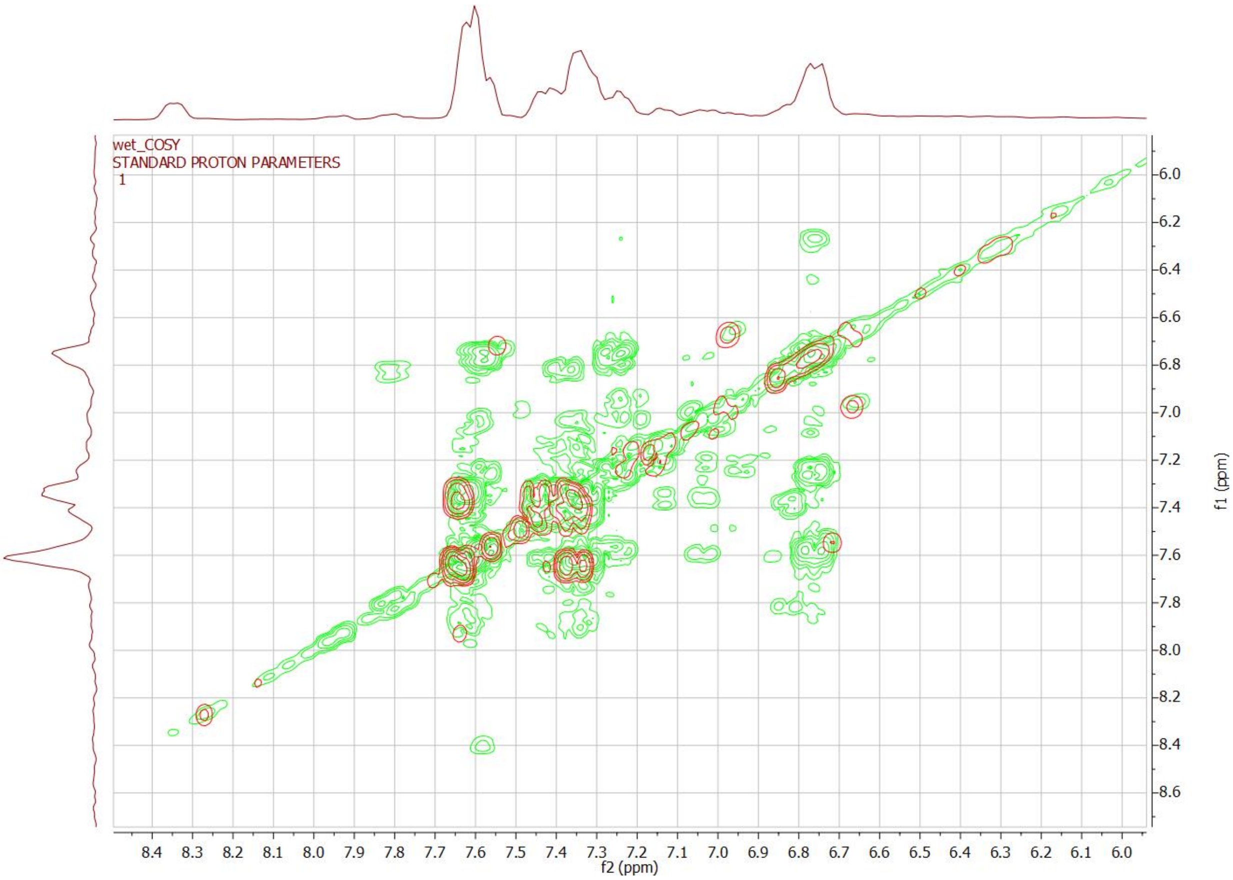

Fig. 4. Comparison of two-dimensional correlation spectra COSY of urine before (red) and after (green) reception of 3g of ASA. Cross peaks are observed in the regions of 7.6, 7.25, 6.75 ppm from the signals of salicylic and salicyrulic acid.

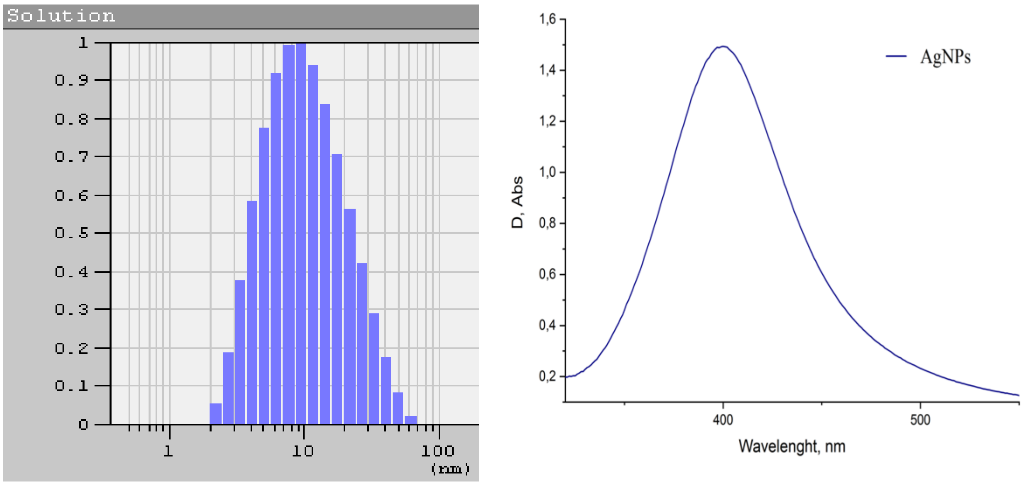

Figure 5. The hydrodynamic radius of the obtained nanoparticles (a) and their absorption spectrum (b) of silver nanoparticles

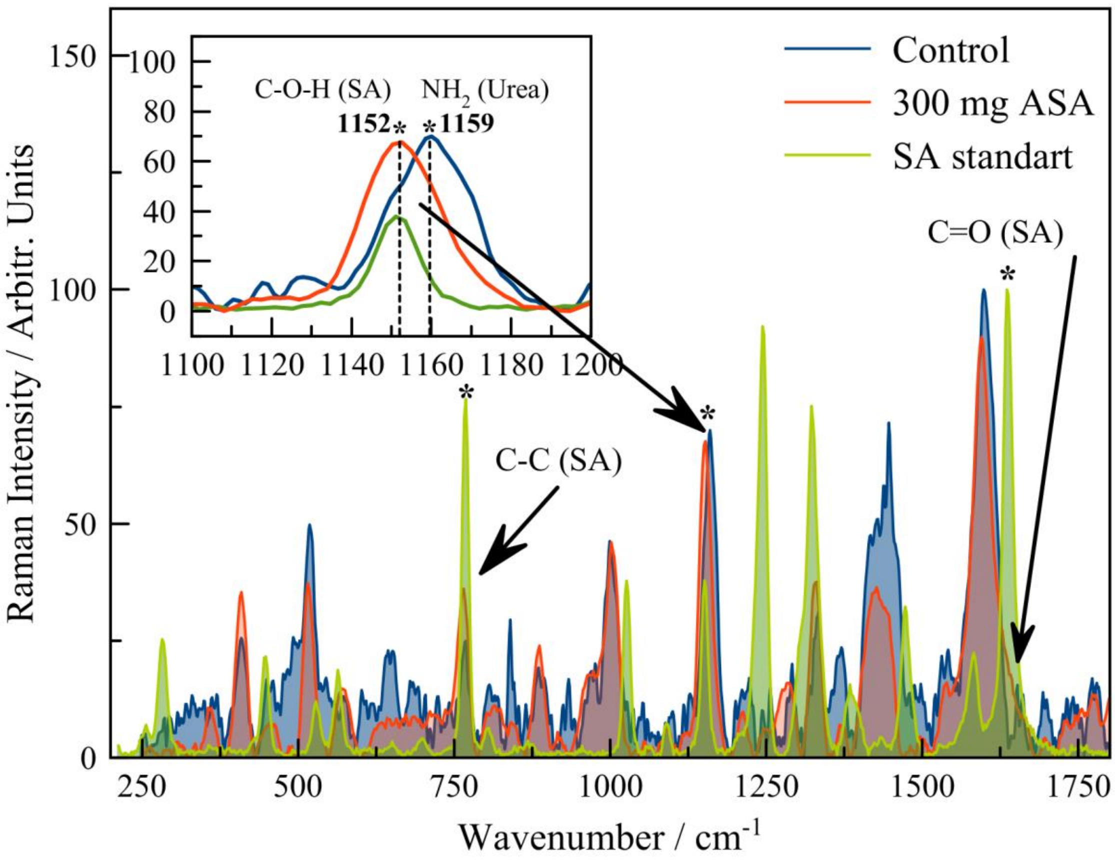

Figure 6. SERS spectra of control (blue line); a urine sample collected after two hours in the case of taking 300 mg of ASA (red line); analytical standard SA (green line). The insert shows the spectral picture in the frequency shift region 1100 - 1200 cm^{-1}

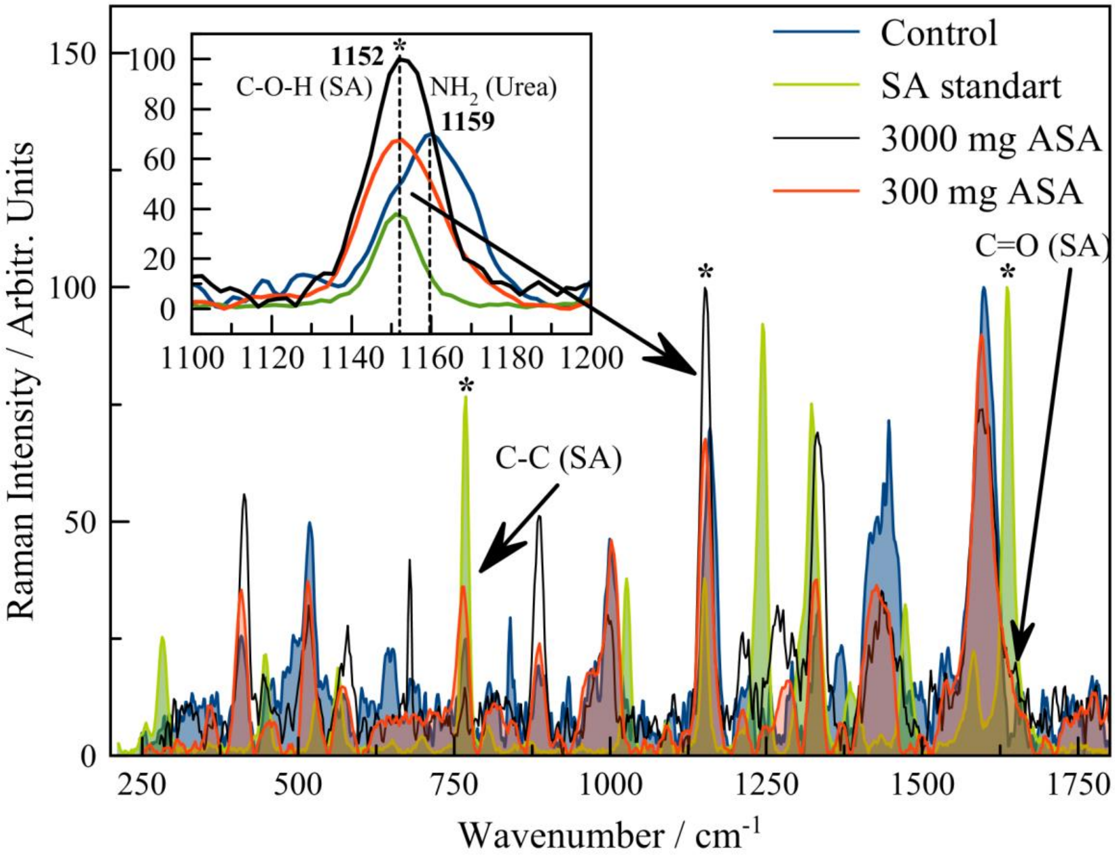

Figure 7. SERS control spectra (blue line); urine sample collected after two hours in case of taking ASA (red line); analytical standard SA (green line); urine sample collected after two hours in case of taking 3000 mg of ASA (black line). The insert shows the spectral picture in the frequency shift region 1100 - 1200 cm^{-1}

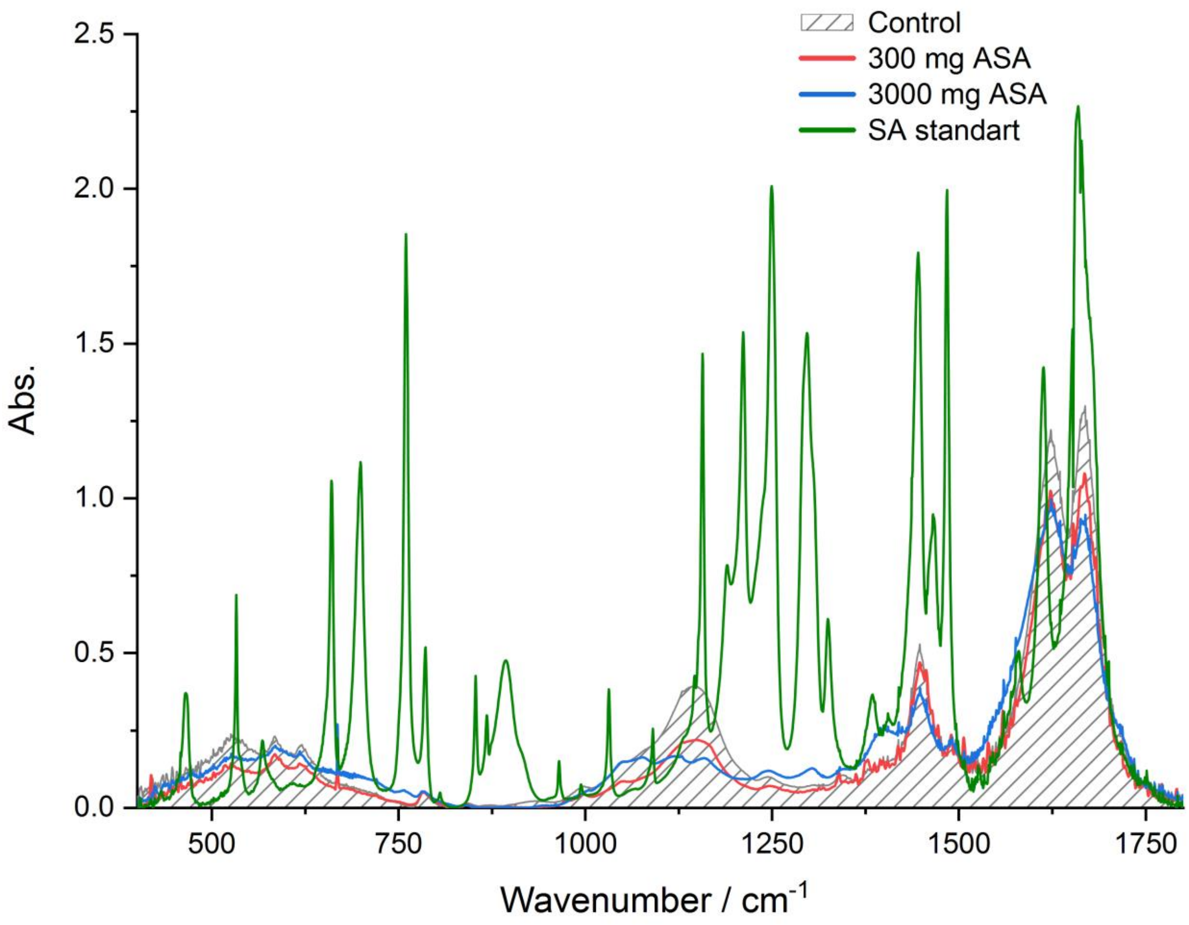

Figure 8. IR Fourier control spectra (hatching); urine sample collected after two hours in the case of taking 300 mg of ASA (red line); analytical standard SA (green line); urine sample collected after two hours in the case of taking 3000 mg of ASA (blue line).

References

1. Mensah GA, Roth GA, Fuster V. The Global Burden of Cardiovascular Diseases and Risk Factors: 2020 and Beyond. Journal of the American College of Cardiology. 2019;74(20):2529-32.
2. Disease GBD, Injury I, Prevalence C. Global, regional, and national incidence, prevalence, and years lived with disability for 354 diseases and injuries for 195 countries and territories, 1990-2017: a systematic analysis for the Global Burden of Disease Study 2017. Lancet. 2018;392(10159):1789-858.
3. Packard KA, Hilleman DE. Adherence to therapies for secondary prevention of cardiovascular disease: a focus on aspirin. Cardiovascular therapeutics. 2016;34(6):415-22.
4. Hankey GJ, Eikelboom JW. Aspirin resistance. Lancet. 2006;367(9510):606-17.
5. Navaratnam K, Alfirevic Z, Pirmohamed M, Alfirevic A. How important is aspirin adherence when evaluating effectiveness of low-dose aspirin? Eur J Obstet Gynecol Reprod Biol. 2017;219:1-9.
6. Hutt AJ, Caldwell J, Smith RL. The metabolism of aspirin in man: a population study. Xenobiotica. 1986;16(3):239-49.
7. Pedersen AK, FitzGerald GA. Dose-related kinetics of aspirin. Presystemic acetylation of platelet cyclooxygenase. N Engl J Med. 1984;311(19):1206-11.
8. Bochner F, Siebert DM, Rodgers SE, McIntosh GH, James MJ, Lloyd JV. Measurement of aspirin concentrations in portal and systemic blood in pigs: effect on platelet aggregation, thromboxane and prostacyclin production. Thromb Haemost. 1989;61(2):211-6.
9. Navarro SL, Saracino MR, Makar KW, Thomas SS, Li L, Zheng Y, et al. Determinants of aspirin metabolism in healthy men and women: effects of dietary inducers of UDP-glucuronosyltransferases. J Nutrigenet Nutrigenomics. 2011;4(2):110-8.
10. Li JP, Guo JM, Shang EX, Zhu ZH, Liu Y, Zhao BC, et al. Quantitative determination of five metabolites of aspirin by UHPLC-MS/MS coupled with enzymatic reaction and its application to evaluate the effects of aspirin dosage on the metabolic profile. J Pharm Biomed Anal. 2017;138:109-17.
11. Liu CH, Das BB, Sha Glassman WL, Tang GC, Yoo KM, Zhu HR, et al. Raman, fluorescence, and time-resolved light scattering as optical diagnostic techniques to separate diseased and normal biomedical media. J Photochem Photobiol B. 1992;16(2):187-209.
12. Hoccart X, Turrell G. Raman spectroscopic investigation of the dynamics of urea–water complexes. The Journal of Chemical Physics. 1993;99(11):8498-503.
13. Frost RL, Kristof J, Rintoul L, Kloprogge JT. Raman spectroscopy of urea and urea-intercalated kaolinites at 77 K. Spectrochim Acta A Mol Biomol Spectrosc. 2000;56A(9):1681-91.
14. Keuleers R, Desseyn HO, Rousseau B, Van Alsenoy C. Vibrational Analysis of Urea. The Journal of Physical Chemistry A. 1999;103(24):4621-30.
15. Lee SH, Jun BH. Silver Nanoparticles: Synthesis and Application for Nanomedicine. Int J Mol Sci. 2019;20(4).
16. Pilot, Signorini, Durante, Orian, Bhamidipati, Fabris. A Review on Surface-Enhanced Raman Scattering. Biosensors. 2019;9(2).
17. Martens J, Koppen V, Berden G, Cuyckens F, Oomens J. Combined Liquid Chromatography-Infrared Ion Spectroscopy for Identification of Regioisomeric Drug Metabolites. Anal Chem. 2017;89(8):4359-62.
18. Barry JA, Groseclose MR, Robichaud G, Castellino S, Muddiman DC. Assessing drug and metabolite detection in liver tissue by UV-MALDI and IR-MALDESI mass spectrometry imaging coupled to FT-ICR MS. Int J Mass Spectrom. 2015;377:448-155.
19. Mendy VL, Vargas R, Zhang L. Aspirin for Prevention of Cardiovascular Disease. Preventing chronic disease. 2017;14:E77.
20. Burns RB, Graham K, Sawhney MS, Reynolds EE. Should This Patient Receive Aspirin?: Grand Rounds Discussion From Beth Israel Deaconess Medical Center. Ann Intern Med. 2017;167(11):786-93.
21. Huang J, Chen X, Xing H, Chen L, Xie Z, He S, et al. Aspirin and heparin for the prevention of pre-eclampsia: protocol for a systematic review and network meta-analysis. BMJ open. 2019;9(2):e026920.

22. Herlitz J, Toth PP, Naesdal J. Low-dose aspirin therapy for cardiovascular prevention: quantification and consequences of poor compliance or discontinuation. Am J Cardiovasc Drugs. 2010;10(2):125-41.
23. Sundstrom J, Hedberg J, Thuresson M, Aarskog P, Johannesen KM, Oldgren J. Low-Dose Aspirin Discontinuation and Risk of Cardiovascular Events: A Swedish Nationwide, Population-Based Cohort Study. Circulation. 2017;136(13):1183-92.
24. Le Gall G. NMR spectroscopy of biofluids and extracts. Methods in molecular biology. 2015;1277:29-36.
25. Kolokolova TN, Sergeev NM, Korol'kov AY. Quantitative determination of metabolites in human urine by NMR 1H spectroscopy. Biomedical Chemistry. 2008;54(2):223-35.
26. Maschke S, Azaroual N, Imbenotte M, Vermeersch G, Leclerc F, Lhermitte M. Salicylate poisoning: two-dimensional J-resolved NMR urinalysis. NMR Biomed. 1995;8(1):19-24.
27. Hoffman MK. Investigating the role of aspirin resistance in obstetrics may optimise prevention of pre-eclampsia. BJOG: An International Journal of Obstetrics & Gynaecology. 2016;123(9):1488-.
28. Rastogi V, Doshi S, Kaleem A. Minimal Change Disease Associated with High-dose Aspirin. Cureus. 2018;10(10):e3408.
29. Ejaz P, Bhojani K, Joshi VR. NSAIDs and kidney. J Assoc Physicians India. 2004;52:632-40.
30. Gambaro G, Perazella MA. Adverse renal effects of anti-inflammatory agents: evaluation of selective and nonselective cyclooxygenase inhibitors. J Intern Med. 2003;253(6):643-52.
31. Lawrence JR, Peter R, Baxter GJ, Robson J, Graham AB, Paterson JR. Urinary excretion of salicyluric and salicylic acids by non-vegetarians, vegetarians, and patients taking low dose aspirin. Journal of clinical pathology. 2003;56(9):651-3.
32. Sathyavathi R, Dingari NC, Barman I, Prasad PS, Prabhakar S, Narayana Rao D, et al. Raman spectroscopy provides a powerful, rapid diagnostic tool for the detection of tuberculous meningitis in ex vivo cerebrospinal fluid samples. J Biophotonics. 2013;6(8):567-72.
33. Bilal M, Saleem M, Amanat ST, Shakoor HA, Rashid R, Mahmood A, et al. Optical diagnosis of malaria infection in human plasma using Raman spectroscopy. J Biomed Opt. 2015;20(1):017002.
34. Rossi EE, Pinheiro ALB, Baltatu OC, Pacheco MTT, Silveira L. Differential diagnosis between experimental endophthalmitis and uveitis in vitreous with Raman spectroscopy and principal components analysis. Journal of Photochemistry and Photobiology B: Biology. 2012;107:73-8.
35. Saatkamp CJ, de Almeida ML, Bispo JA, Pinheiro AL, Fernandes AB, Silveira L, Jr. Quantifying creatinine and urea in human urine through Raman spectroscopy aiming at diagnosis of kidney disease. J Biomed Opt. 2016;21(3):37001.
36. Bispo JA, de Sousa Vieira EE, Silveira L, Jr., Fernandes AB. Correlating the amount of urea, creatinine, and glucose in urine from patients with diabetes mellitus and hypertension with the risk of developing renal lesions by means of Raman spectroscopy and principal component analysis. J Biomed Opt. 2013;18(8):87004.
37. Moreira LP, Silveira L, Jr., da Silva AG, Fernandes AB, Pacheco MTT, Rocco D. Raman spectroscopy applied to identify metabolites in urine of physically active subjects. J Photochem Photobiol B. 2017;176:92-9.
38. Bonifacio A, Cervo S, Sergo V. Label-free surface-enhanced Raman spectroscopy of biofluids: fundamental aspects and diagnostic applications. Anal Bioanal Chem. 2015;407(27):8265-77.
39. Izquierdo-Lorenzo I, Alda I, Sanchez-Cortes S, Garcia-Ramos JV. Adsorption and Detection of Sport Doping Drugs on Metallic Plasmonic Nanoparticles of Different Morphology. Langmuir. 2012;28(24):8891-901.
40. Izquierdo-Lorenzo I, García-Ramos JV, Sanchez-Cortes S. Vibrational characterization and surface-enhanced Raman scattering detection of probenecid doping drug. Journal of Raman Spectroscopy. 2013;44(10):1422-7.
41. Kao Y-C, Han X, Lee YH, Lee HK, Phan-Quang GC, Lay CL, et al. Multiplex Surface-Enhanced Raman Scattering Identification and Quantification of Urine Metabolites in Patient Samples within 30 min. ACS Nano. 2020;14(2):2542-52.
42. van Outersterp RE, Houthuijs KJ, Berden G, Engelke UF, Kluijtmans LAJ, Wevers RA, et al. Reference-standard free metabolite identification using infrared ion spectroscopy. International Journal of Mass Spectrometry. 2019;443:77-85.

43. Lafrance D. Measurement of lactate in whole human blood with near-infrared transmission spectroscopy. Talanta. 2003;60(4):635-41.
44. Nakamura T, Takeuchi T, Terada A, Tando Y, Suda T. Near-Infrared Spectrometry Analysis of Fat, Neutral Sterols, Bile Acids, and Short-Chain Fatty Acids in the Feces of Patients with Pancreatic Maldigestion and Malabsorption. International Journal of Gastrointestinal Cancer. 1998;23(2):137-44.
