# Supplementary material for: NMR spectroscopy reveals acetylsalicylic acid metabolites in the human urine for drug compliance monitoring
Source: PLoS One. 2021 Mar 8;16(3):e0247102. doi: 10.1371/journal.pone.0247102 (PMC7939264; doi:10.1371/journal.pone.0247102)
Supplement: Attachment — Submitted filename: Response to Reviewer 1.docx [file pone.0247102.s002.docx]

Response to Reviewer #1

We are grateful to the reviewer for careful reading of the manuscript and for useful expert comments. We have modified the manuscript according to all the reviewer comments.

*Comments for author*

*Reviewers #1*

*1. The authors failed to prove applicability of the methods (SERS and IR in particularly) for the determination of ASA metabolites in urine*

*The paper seems more like a compilation of poorly performed single experiments than the comprehensive study.*

Authors’ response:

The authors have removed the part concerning the applicability of the methods (SERS and IR).

*2. The authors write: «The NMR method was sensitive to the detection of low concentrations of ASA metabolites in the urine (at a dosage of 100 mg)». However, quantitative information about concentrations of the metabolites in urine absents within whole paper, i.e., the relationship between metabolite concentration in urine and dosage (or, more importantly, therapeutical effect) has not been demonstrated and proved.*

Authors’ response:

In our work, we detected ^1^Н NMR signals of the main metabolites of aspirin (ASA) in urine samples of volunteers after taking the drug in therapeutic doses (at a dose of 100 mg). We were primarily interested in the fundamental issue of changing the profile of the NMR spectrum of urine samples and the possibility of identifying markers that can be used to control the intake of the drug in low doses. Such results have not been previously reported in the literature. In the literature, there are some ^1^H NMR data on the detection of metabolites which arise in the urine of patients in cases of salicylic acid poisoning. However, there are a few data on the detection of ASA metabolites after taking the drug at therapeutic doses.

Indeed, the authors do not give quantitative estimates, since the task of quantitative analysis was not posed at this stage. The solution to this problem requires special attention. As a rule, quantitative estimates are made based on a comparative analysis using a reference signal. Thus, the authors (Tuulia Tynkkynen and all. Proof of concept for quantitative urine NMR metabolomics pipeline for large-scale epidemiology and genetics International Journal of Epidemiology, 2019, 978–993) apply a standard protocol to normalize creatinine levels. They suggested making estimates of the intensity relative to the creatine signal. However, in our research, we found that the intensity of creatinine signals also changes. Other authors (Nicolescu A., Simionescu N., Ursu L., Simionescu *B.* The effect of therapeutic doses of paracetamol and asperin of the NMR profile of urine at 400MHz. Rev. Roum. Chim.,2012, *57*(7-8), 653-658) quantified SU in urine samples using signals corresponding to protons from 7.78 ppm. This is one of the signals of the aromatic group of SU. This approach is also incorrect, since we are dealing with an interacting system of proton spins. Moreover, the signals of metabolites in the aromatic region overlap with other signals. These facts also make it difficult to quantify correctly. We are now developing a new approach to solving this problem and hope to find a solution.

*3. The reproducibility and accuracy of the results were not investigated as well. This is a significant disadvantage if to account highly deviating composition of the urine and, consequently, its deviating background signal.*

Authors' response:

The reviewer rightly points out the need and importance of reproducibility and accuracy assessments. The authors conducted additional studies of the volunteers' urine. In order to assess reproducibility and accuracy, the number of samples tested was increased, and a comparative analysis of urine samples collected at different times of the year was carried out.

The manuscript contains edits.

*4. The results in Fig. 7 and 8 are failed to confirm the presence of SA in the urine samples because the spectra of contaminated samples are too different compared to the spectrum of SA standard.*

Authors’ response:

Research results related to IR and Raman scattering (the results in Fig. 7 and 8) are removed from the article.

*5. The paper lacks an adequate interference study. Although the authors know that urine is a complex mixture, they estimated only contribution of urea to the signal. However, there are numerous other analytes which can contribute to the final SERS signal, e.g., creatinine, uric acid, various metabolites of hemoglobin (e.g., urobilin), etc. All these components possess large Raman cross-section and can generate significant background signal making determination of ASA metabolites impossible.*

Authors’ response:

Research results related to IR and Raman scattering are removed from the manuscript

*6 The authors should clearly formulate why someone need to determine SA and SU in urine.*

Authors’ response:

The main goal of the study is to find markers that can be used to monitor aspirin intake. Since SA and SU are the most important metabolites of aspirin, the emergence of SA and SU in urine. This is one of the important indicators of aspirin intake. However, it should be noted that a number of interesting effects were also observed in the urine images of patients after taking the drug, such as a change in the signal profile of urea and hippuric acid. These effects are still awaiting investigation and explanation. exist although still discovered

The manuscript has been supplemented to clarify the main goal of the study and the need to detect SA and SU.

*7. The level of English is sometimes quite poor. Also, there are numerous typos.*

Authors’ response:

The authors have removed typos in the text.

*8. The advantages and limitation of the methods compared to other well-known methods should be analyzed and discussed.*

Authors’ response:

The advantages and limitations of the NMR method were analyzed in comparison with other known methods.

*9. The authors should select only one of the used methods and perform really comprehensive study collecting appropriate value of statistical data under supervision of qualified analytical chemist.*

Authors’ response:

The authors focused their attention on the NMR method and carried out an additional study of the samples.

The article is supplemented with the Discussion section.
